# Effect of Theta Burst Stimulation-Patterned rTMS on Motor and Nonmotor Dysfunction of Parkinson's Disease: A Systematic Review and Metaanalysis

**DOI:** 10.3389/fneur.2021.762100

**Published:** 2022-01-12

**Authors:** Bo Cheng, Tao Zhu, Wenhao Zhao, Ling Sun, Yao Shen, Wei Xiao, Shushan Zhang

**Affiliations:** ^1^Department of Neurology, Affiliated Hospital of Medical College, North Sichuan Medical College (University), Nanchong, China; ^2^Department of Preventive Medicine, North Sichuan Medical College (University), Nanchong, China

**Keywords:** Parkinson's disease, repetitive transcranial magnetic stimulation, theta burst stimulation, non-invasive brain stimulation, meta-analysis

## Abstract

**Background:** Theta burst stimulation (TBS), a type of patterned repetitive transcranial magnetic stimulation (rTMS), has several advantages, such as short time of single treatment and low stimulation intensity compared with traditional rTMS. Since the efficacy of TBS on the symptoms of Parkinson's disease (PD) was inconsistent among different studies, we systematically searched these studies and quantitatively analyzed the therapeutic effect of TBS for patients with PD.

**Methods:** We followed the recommended PRISMA guidelines for systematic reviews. Studies from PubMed, EMBASE, CENTRAL, and ClinicalTrials.gov from January 1, 2005 of each database to September 30, 2021 were analyzed. We also manually retrieved studies of reference.

**Results:** Eight eligible studies with 189 participants (received real TBS and/or sham TBS) were included. This metaanalysis found that TBS did not significantly improve Unified Parkinson's Disease Rating Scale part III (UPDRS-III) score in the “on” medicine state (SMD = −0.06; 95% CI, −0.37 to 0.25; *p* = 0.69; *I*^2^ = 0%), while, it brought significant improvement of UPDRS-III scores in the “off” medicine state (SMD = −0.37; 95% CI, −0.65 to −0.09; *p* < 0.01; *I*^2^ = 19%). Subgroup analysis found that merely continuous TBS (cTBS) over the supplementary motor area (SMA) brought significant improvement of UPDRS-III score (SMD = −0.63; 95% CI, −1.02 to −0.25; *p* < 0.01). TBS had insignificant effectiveness for upper limb movement disorder both in the “on” and “off” medicine status (SMD = −0.07; 95% CI, −0.36 to 0.22; *p* = 0.64; *I*^2^ = 0%; SMD = −0.21; 95% CI, −0.57 to 0.15; *p* = 0.26; *I*^2^ = 0%; respectively). TBS significantly improved slowing of gait in the “off” medicine status (SMD = −0.37; 95% CI, −0.71 to −0.03; *p* = 0.03; *I*^2^ = 0%). Subgroup analysis suggested that only intermittent TBS (iTBS) over the primary motor cortex (M1) + dorsolateral prefrontal cortex (DLPFC) had significant difference (SMD = −0.57; 95% CI, −1.13 to −0.01; *p* = 0.04). Additionally, iTBS over the M1+ DLPFC had a short-term (within 2 weeks) therapeutic effect on PD depression (MD = −2.93; 95% CI, −5.52 to −0.33; *p* = 0.03).

**Conclusion:** Our study demonstrated that cTBS over the SMA could significantly improve the UPDRS-III score for PD patients in the “off,” not in the “on,” medicine state. TBS could not bring significant improvement of upper limb movement dysfunction. ITBS over the M1+DLPFC could significantly improve the slowing of gait in the “off” medicine status. Additionally, iTBS over the M1+DLPFC has a short-term (within 2 weeks) therapeutic effect on PD depression. Further RCTs of a large sample, and excellent design are needed to confirm our conclusions.

## Introduction

Parkinson's disease (PD) is the second most common neurodegenerative disorder. The prevalence of PD in industrialized countries is generally estimated at 0.3% of the entire population and ~1% of people over 60 years of age (1). The classical motor symptoms of PD include bradykinesia, muscular rigidity, rest tremor, and postural and gait impairment. Nonmotor dysfunction, such as depression, and cognitive impairment are also frequently present (2). PD is associated with functional deficits in multiple brain areas, including basal ganglia nuclei, cerebellum, and cortical areas (3).

Transcranial magnetic stimulation (TMS) is a non-invasive and painless method to stimulate the human brain (4). Repetitive TMS (rTMS) refers to the application of trains of regularly repeating TMS pulses. Repeated applications of it can produce even long-term effects that last for weeks to months (4–6). In addition to the local stimulatory effect on the cortical area, rTMS can also induce a distant effect on other cortical and subcortical brain regions, probably *via* the cortico-basal, ganglia–thalamocortical motor circuit (7, 8). The previous work showed that high-frequency (HF) rTMS targeting bilateral primary motor cortex (M1) regions could improve the motor performance in patients with PD (9–14), and HF rTMS of left dorsolateral prefrontal cortex (DLPFC) had an antidepressant effect on patients with PD (13, 15, 16). Relevant evidence-based guidelines also gave recommendations on the therapeutic effect of rTMS in motor symptoms and depression in patients with PD (17).

Theta burst stimulation (TBS), a variation of rTMS, may facilitate induction of plasticity mechanisms (18), which affords a short stimulation duration, low stimulation pulse intensity, and a possibility to improve rTMS efficiency (19). When TBS is delivered continuously (cTBS), it decreases cortical excitability, whereas intermittent TBS (iTBS) increases cortical excitability (20). Since TBS may have fewer adverse effects, such as seizures, impairment of hearing and cognition function (21), and shorter time of single intervention (within several minutes) compared with traditional rTMS, in recent years, an increasing number of studies have begun to explore the therapeutic effect of TBS on the motor and nonmotor symptoms in patients with PD (22–35). Nevertheless, there are inconsistencies of conclusion among these studies. Besides, the latest evidence-based guidelines did not include recommendations on the therapeutic effect of TBS in patients with PD (17), which brought dilemma to clinical practice. This systematic review and metaanalysis examined the studies on the therapeutic effect and tolerability of TBS for motor and nonmotor dysfunction in patients with PD.

## Methods

### Study Design

Our meta-analysis is according to the Preferred Reporting Items for Systematic Reviews and Meta-analyses (PRISMA) statement (36).

### Study Search and Selection

We systematically retrieved relevant literature published in PubMed, EMBASE, and CENTRAL, and ClinicalTrials.gov from January 1, 2005 to September 30, 2021. Database searches were limited to articles published in English. Besides, we also manually retrieved studies of reference. The search terms we used to query the databases were as follows: (“Parkinson Disease” or “Parkinson's Disease” or “Parkinsonism”) and (“theta-burst stimulation” or “TBS” or “repetitive transcranial magnetic stimulation” or “rTMS” or “non-invasive stimulation)”. Studies were included if they met the “PICOS” as follows: population [patients with a diagnosis of idiopathic PD according to the UK PD Brain Bank criteria, (37)], intervention (received true patterned rTMS: TBS), comparators (sham TBS), outcome measure (clinical evaluation of motor and nonmotor symptoms), and study design (clinical trial). Studies were excluded if: (1) they were clear from the article title or abstract that did not meet the inclusion criteria, (2) they did not have data available (mean ± SD/SE) for effect size estimation or lacked sufficient reporting detail, such as conference abstract or presentation review articles, editorials, and other nonclinical trials.

Two investigators (BC and TZ) independently screened the titles and abstracts of the literature and decided whether the article should be further retrieved according to the inclusion and exclusion criteria. Those that could not be excluded were retrieved, and the full text was reviewed by the two reviewers (WZ and LS). For articles that may be included, if reported data were insufficient for data analysis, we contacted the corresponding author by email to request access to additional data. Any disagreements were resolved by discussion with a third reviewer (SZ).

### Data Extraction

The following information was extracted by two reviewers (BC and TZ) independently, from included studies: first author, year of publication, study design, patients' age, sample size, PD duration, gender distribution, Hoehn and Yahr scale, timepoints of assessment, neuropsychological symptom assessment scale, TBS protocols, and study quality.

### Quality Assessment

The quality assessments were performed with the PEDro scale (38), which is based on the Delphi List criteria (39) and is considered valid and reliable (38, 40). The PEDro scale assesses the methodological quality of a study based on important criteria, such as concealed allocation, intention-to-treat analysis, and the adequacy of follow-up. These characteristics make the PEDro scale a useful tool for assessing the quality of physical therapy and rehabilitation trials. The PEDro scale consisted of 11 items. One item on the PEDro scale (eligibility criteria) is related to external validity and is generally not used to calculate the method score (41, 42). Therefore, a score of 0–10 was allocated to each study (9–10: excellent; 6–8: good; 4–5: fair; and ≤ 3: poor) (43). Additionally, publication bias on included studies was assessed by the funnel plot and bias tests. If the plot is symmetrical or *p* > 0.05 from bias tests, it should be deemed not publication bias.

### Data Synthesis and Statistical Analysis

The RevMan5.3 and Stata16 software were used to combine the data when at least two studies reported similar clinical outcomes. For quantitative synthesis, the effect size was calculated based on the mean prepost change in the treatment group minus the mean prepost change in the comparison group, divided by the pooled pretest standard deviation (44). If the unit of measurement was consistent across trials, the results were presented as the weighted mean difference (MD) with 95% confidence intervals (CIs) or else replaced by the standard mean difference (SMD). Data from the standard error of the mean (SEM) were converted to the standard deviation (SD) using sample size in the formula SD = SEM × √N (45). We used the random-effects model and fixed-effects model to calculate the pooled SMD and generated forest plots to display the single study and pooled-effect size. The Chi-square test and *I*^2^ statistic were used to assess heterogeneity among studies. Heterogeneity was considered significant if the *p*-value of the χ^2^ test was < 0.1 or *I*^2^ > 50% (46–48). If there was no significant heterogeneity, the fixed-effects model was used to pool data across the included studies; otherwise, the random-effects model was used (49, 50).

## Results

### Search Results

**Figure 1:**
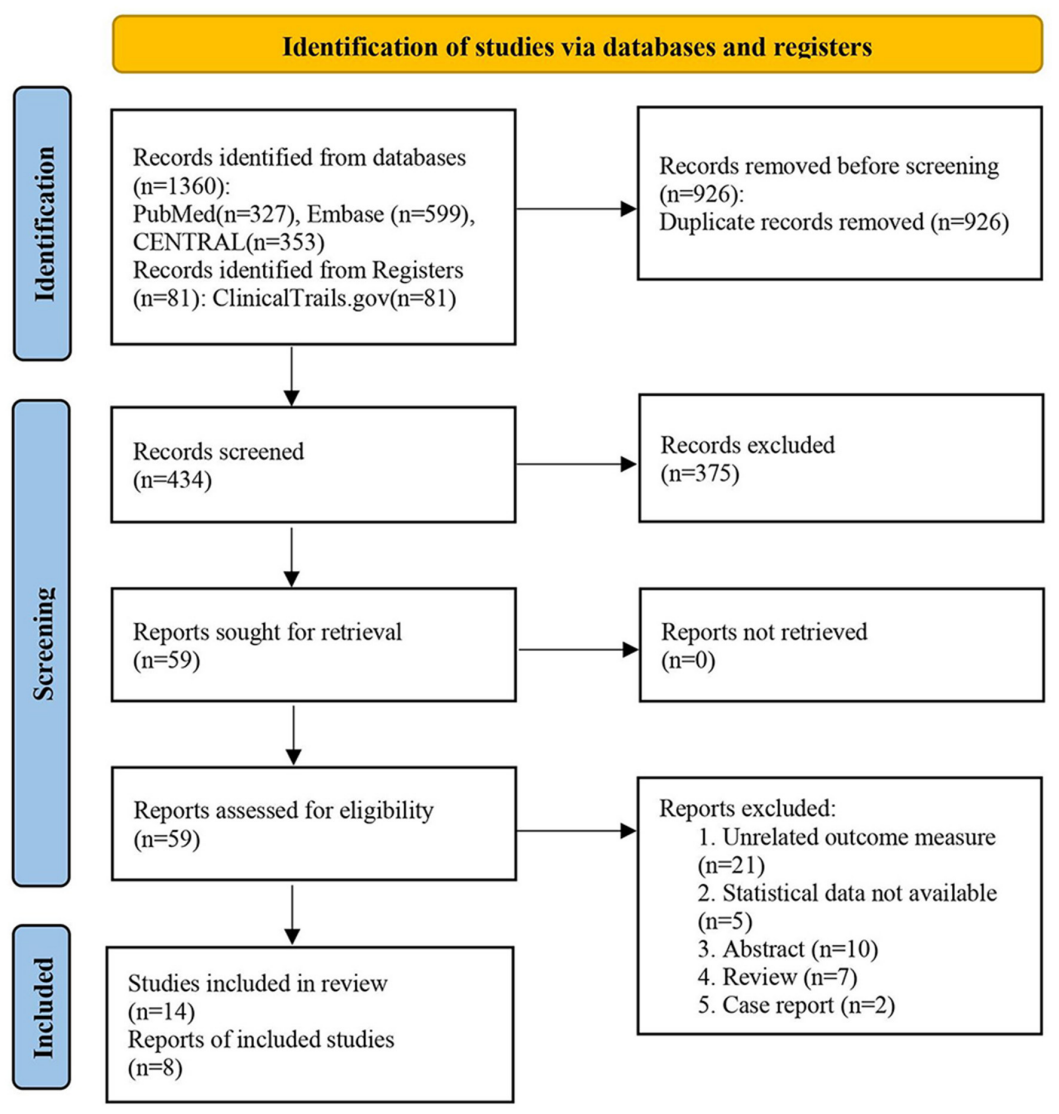
PRISMA search strategy flow diagram of the studies selection process.

Our search strategy to query limited databases retrieved 1,360 studies, while, many of these were identified as duplicates. After screening the titles and abstracts, 375 records were excluded as they did not meet the inclusion criteria (“PICOS”) in our work, and we determined 59 articles for full-text reading. Finally, eight studies were included in our metaanalysis (23–25, 28–30, 32, 34). The literature selection is presented in Figure 1.

### Study Characteristics

Eight eligible studies included for metaanalysis had 189 participants (received real TBS and/or sham TBS).

Four studies (24, 25, 30, 32) were parallel-controlled and four studies (23, 28, 29, 34) were crossover-controlled. Five studies gave single-session (23, 25, 28, 29, 34) and three studies gave multiple sessions (total of six to 42 sessions) (24, 30, 32). Four studies evaluated the immediate effects after TBS using clinical symptom assessment scales (23, 25, 28, 29, 34), and three studies conducted a follow-up of clinical effects range from 1 week to 1 month after TBS intervention (24, 30, 32). Two studies assessed the therapeutic effects of TBS only in the “on” anti-Parkinsonism medicine state (29, 51). While, two studies evaluated only in the “off” medicine status (under the anti-Parkinsonism medicine withdrawal status for at least 12 hours) (23, 34). Besides, four studies assessed the therapeutic effects of TBS both in the “on” and “off” medicine state (24, 25, 28, 32). The characteristics of the included studies are summarized in Table 1, and the TBS intervention parameters are summarized in Table 2.

**Table 1:** Characteristics of included studies.

**Study**	**Design**	**Age, y**	**Sample Size**	**PD** **duration, y**	**Sex (M/F)**	**HandY stage**	**Assessment time (pre-TBS, post-TBS)**	**Outcome measures** **(Clinical assessment)**
Benninger et al. (24)	Parallel	E:62.1 ± 6.9 C:65.6 ± 9.0	E:13 C:13	E:10.8 ± 7.1 C:6.5 ± 3.4	E:7/6 C:11/2	E:2.6 ± 0.2^a^ 3.0 ± 0.4^b^ C:2.5 ± 0.1^a^ 2.9 ± 0.2^b^	Baseline, 1th day, 1th month	Primary: Gait and bradykinesia by measuring UPDRS sub-items Secondary: UPDRS total, UPDRS-III, UPDRS-II, UPDRS- freezing, FAB, BDI, mental health, physical health, SRTT
Bologna et al. (27)	Crossover	61.9 ± 6.0	13	5.3 ± 4.46	9/4	NA	Baseline, 5th, 45th minute	MDS UPDRS-III items 3.17 (resting tremor amplitude)
Brugger et al. (34)	Crossover	64.30 (52.8–68.3) ^c^	12	12.5 (10.5–15.0)^c^	10/2	2.0 (2.0–2.8) ^c^	Baseline, immediately	UPDRS-III
Degardin et al. (25)	Parallel	E1:61.5 ± 8.5^a^ 61.3 ± 9.6^b^ E2:60.6 ± 11.8 C: 61.5 ± 9.9^a^	E1:11 E2:10 C:11	E1:6.8 ± 2.7^a^ 6.2 ± 2.5^b^ E2:1.8 ± 1 C: 8.2 ± 5.2^a^	E1:4/7^a^ 3/5^b^ E2:8/2 C:7/4	E1:2 ± 0.63^a^ 2 ± 0.75^b^ E2:1.3 ± 0.48 C:2.2 ± 0.63^a^	Baseline, immediately	UPDRS-III (finger tapping, hand movement and arm rigidity items from 0 to 4)
Eggers et al. (23)	Crossover	68.5 ± 5	8	4 ± 3	6/2	1.97 ± 0.58	Baseline, immediately	UPDRS-III (items 18–31, maximum: 108 points), PPT
Eggers et al. (28)	Crossover	E:60.8 ± 7.8^a^ 64.7 ± 5.0^b^	E:13^a^ 13^b^	E:7.1 ± 4.7^a^ 5.8 ± 4.3^b^	6/7^a^ 9/4^b^	1.7 ± 0.8^a^ 1.8 ± 0.8^b^	Baseline, immediately	UPDRS-III (items 18–31, maximum: 108 points), PPT
He et al. (35)	Parallel	E:70.0 ± 6.3 C:74.8 ± 6.9	E:20 C:15	E:2.7 ± 1.5 C:2.5 ± 1.1	E:13/7 C:10/5	E:2.7 ± 1.1 C:2.5 ± 1.0	Baseline, immediately, 3rd month	RBANS, MoCA
Hill et al. (31)	Crossover	71.07 ± 5.11	14	4.86 ± 4.85	10/4	NA	Baseline, 5th, 30th min	BCST, N-Back tasks
Ji et al. (32)	Parallel	E: 61.7 ± 1.57 C: 60.2 ± 1.97	E:22 C:20	E: 4.3 ± 0.52 C: 5.3 ± 0.83	E:14/8 C:14/6	E:1.6 ± 0.12 C:1.7 ± 0.11	Baseline, 1st, 2nd week	Primary: UPDRS-III (2 weeks) Secondary: UPDRS-III (1 week), NMSS, timed up-and-go, 20-m walking
Koch et al. (22)	Parallel	64.7 ± 6.9	E:10 C:10	10.4 ± 4.3	NA	NA	Baseline, 2nd, 4th week	Global CAPSIT dyskinesia scale scores, UPDRS-III
Lang et al. (33)	Parallel	E: 68.43 ± 8.4 C:68.76 ± 8.3	E: 21 C: 20	E:5.95 ± 4.8 C:4.8 ± 4.0	E:14/7 C:13/7	NA	Baseline, 1th day, 1th month	Primary: Neuropsychological Tests battery according to five cognitive domains^d^ Secondary: UPDRS-III, BDI-II, BAI
Trung et al. (30)	Parallel	E:71.3 ± 7.3 C:67.3 ± 5.2	E:14 C:14	E:10.39 ± 6.7 C:6.25 ± 3.0	E:8/6 C:11/3	1 to 3	Baseline,1st, 10th, 30th day	Primary: Neuropsychological Test battery according to cognitive domains^e^ Secondary: SETS, BDI, BAI, AES, PDQ-39, UPDRS-III, MoCA
Vanbellingen et al. (29)	Crossover	66 ± 8.10	15	8.24 ± 4.64	11/4	2 ± 0.58	Baseline, immediately	CRT, Mod-MDS-UPDRS III, Jamar, proprioception (specific distal finger)
Zamir et al. (26)	Parallel	E:64.7 ± 10.3 C:63.1 ± 8.8	E:12 C:10	7.3 ± 3.2	E:7/5 C:4/6	NA	Baseline, Immediately	UPDRS-III

*AES, Apathy Evaluation Scale; BAI, Beck Anxiety Inventory; BCST, the Berg's Card Sorting Test; BDI, Beck Depression Inventory; C, control group; CAPSIT, Core Assessment Program for Surgical Interventional Therapies; CRT, Coin rotation task; E, experimental group; FAB, Frontal Assessment Battery; HandY, Hoehn and Yahr scale; MDS, Movement Disorder Society; MoCA, Montreal Cognitive Assessment; NA, not available; NMSS, Non-Motor Syndrome Scale; PD, Parkinson's Disease; PDQ-39, Parkinson Daily Questionnaire-39; PPT, Purdue Pegboard Test; RBANS, Repeatable Battery for the Assessment of Neuropsychological Status; SETS, Stanford Expectations of Treatment Scale; SRTT, Serial Reaction Time Task; TBS, theta-burst stimulation; UPDRS, Unified Parkinson's Disease Rating Scale (part II, activities of daily living; part III, motor examination; freezing, UPDRS II, item 14, freezing when walking); VAS, visual analog scales.*

*^a^“on” anti-Parkinsonism medicine.*

*^b^“off” anti-Parkinsonism medicine.*

*^c^data are reported as medians (interquartile range).*

*^d^including Stroop Color and Word Test, Brixton Spatial Anticipation, Hayling Sentence Completion Section Methods, Trail Making Test (B), Clock Drawing Test (Command), Trail Making Test (A), Wechsler Memory Scale -IV Symbol Span, Wechsler Adult Intelligence Scale -IV Digit Span (Forward), Boston Naming Test, Semantic Fluency (Animals/Actions), Benton Judgement of Line Orientation, Rey Complex Figure Copy Test copy trial, Hopkins Verbal Learning Test (Retention/Discrimination Index), Wechsler Memory Scale -IV Logical Memory, Rey Complex Figure Copy Test delayed recall trial.*

*^e^including Trail Making Test Part A, Digit span test (forward, backward), Digit symbol test, Stroop test (color scale, naming scale), Brixton, Montreal Evaluation of Communication protocol test (orthographic verbal fluency), Trail Making Test Part B, Stroop test (inhibition scale), Boston Naming, Montreal Evaluation of Communication protocol test (semantic verbal fluency, without constraint verbal fluency), Rey-Osterrieth test (immediate recall), Rey Auditory Verbal Learning Test (RAVLT 1,2,3,4,5,1 to 5, Interference, RAVLT 6, Delay, Recognition), Hooper Visual Organization Test, Rey-Osterrieth test (figure copy), alternating with Taylor test (figure copy), Clock-drawing subtest of the Montreal Cognitive Assessment Scale*.

**Table 2:** Study characteristics of TBS protocols included in the meta-analysis.

**Study**	**Treatment protocol**	**Frequency**	**Intensity**	**Coil-type**	**Brain target**	**Continuous/** **discontinuous days**	**Session(s)/ duration**	**Pulses/** **Session**
Benninger et al. (24)	iTBS	50Hz	80%AMT	C	DLPFC+M1 (bilateral)	Continuous (4 days)	8/2weeks	600
Brugger et al. (34)	iTBS	50Hz	100%AMT	F8	SMC (bilateral)	_	Single-session	600
Degardin et al. (25)	iTBS	50Hz	80%AMT	F8	M1	_	Single-session	600
Eggers et al. (23)	cTBS	50Hz	80%AMT	F8	M1	_	Single-session	600
Eggers et al. (28)	cTBS	50Hz	90%AMT	F8	SMA	_	Single-session	600
Ji et al. (32)	cTBS	50Hz	80%RMT	F8	SMA (left)	Continuous (14 days)	42/14days	600
Trung et al. (30)	iTBS	50Hz	80%AMT	F8	DLPFC (left)	Discontinuous	6/1week (within)	600
Vanbellingen et al. (29)	iTBS cTBS	30Hz 30Hz	80%RMT 80%RMT	F8 F8	PMd PMd	_ _	Single-session Single-session	801 801

*AMT, active motor threshold; C, circular; cTBS, continuous theta burst stimulation; DLPFC, dorsolateral prefrontal cortex; F8, figure of 8; iTBS, intermittent theta-burst stimulation; M1, primary motor cortex; PMd, dorsal pre-motor cortex; RMT, resting motor threshold; SMA, supplementary motor area; SMC, supplementary motor cortex*.

### Quality Assessment

The PEDro scores of the included studies ranged from 6 to 9, with mean scores of 7. Two studies were of excellent quality (24, 32) and five studies were of good quality. No studies (23, 25, 28–30, 34) were assessed as fair quality or poor quality. A detailed evaluation of the methodological quality of included studies for metaanalysis is provided in Table 3. Egger's test by Stata16 showed no significant publication bias for all clinical symptoms of quantitative analysis.

**Table 3:** Quality assessment of the studies included in the meta-analysis.

**Study**	**1**	**2**	**3**	**4**	**5**	**6**	**7**	**8**	**9**	**10**	**11**	**Total** **score**
Benninger et al. (24)	Y	Y	Y	Y	Y	N	Y	Y	Y	Y	Y	9
Brugger et al. (34)	Y	Y	N	Y	Y	N	N	Y	Y	Y	Y	7
Degardin et al. (25)	Y	N	N	Y	Y	N	Y	N	Y	Y	Y	6
Eggers et al. (23)	Y	Y	N	Y	Y	N	N	N	Y	Y	Y	6
Eggers et al. (28)	Y	Y	N	Y	Y	N	N	N	Y	Y	Y	6
Ji et al. (32)	Y	Y	Y	Y	Y	N	Y	Y	Y	Y	Y	9
Trung et al. (30)	Y	Y	N	Y	Y	N	N	Y	Y	Y	Y	7
Vanbellingen et al. (29)	Y	N	N	Y	Y	N	Y	N	Y	Y	Y	6
Mean												7

1, Eligibility criteria and source of participants; 2, Random allocation; 3, Concealed allocation; 4, Baseline comparability; 5, Participant blinding; 6, Therapist blinding; 7, Assessor blinding; 8, Adequate follow-up; 9, Intention-to-treat analysis; 10, Between group comparison; 11, Point estimates and variability; ^*^ Item 1 dose not contribute to the total score.

### Effects of TBS on UPDRS-III Score

**Figure 2:**
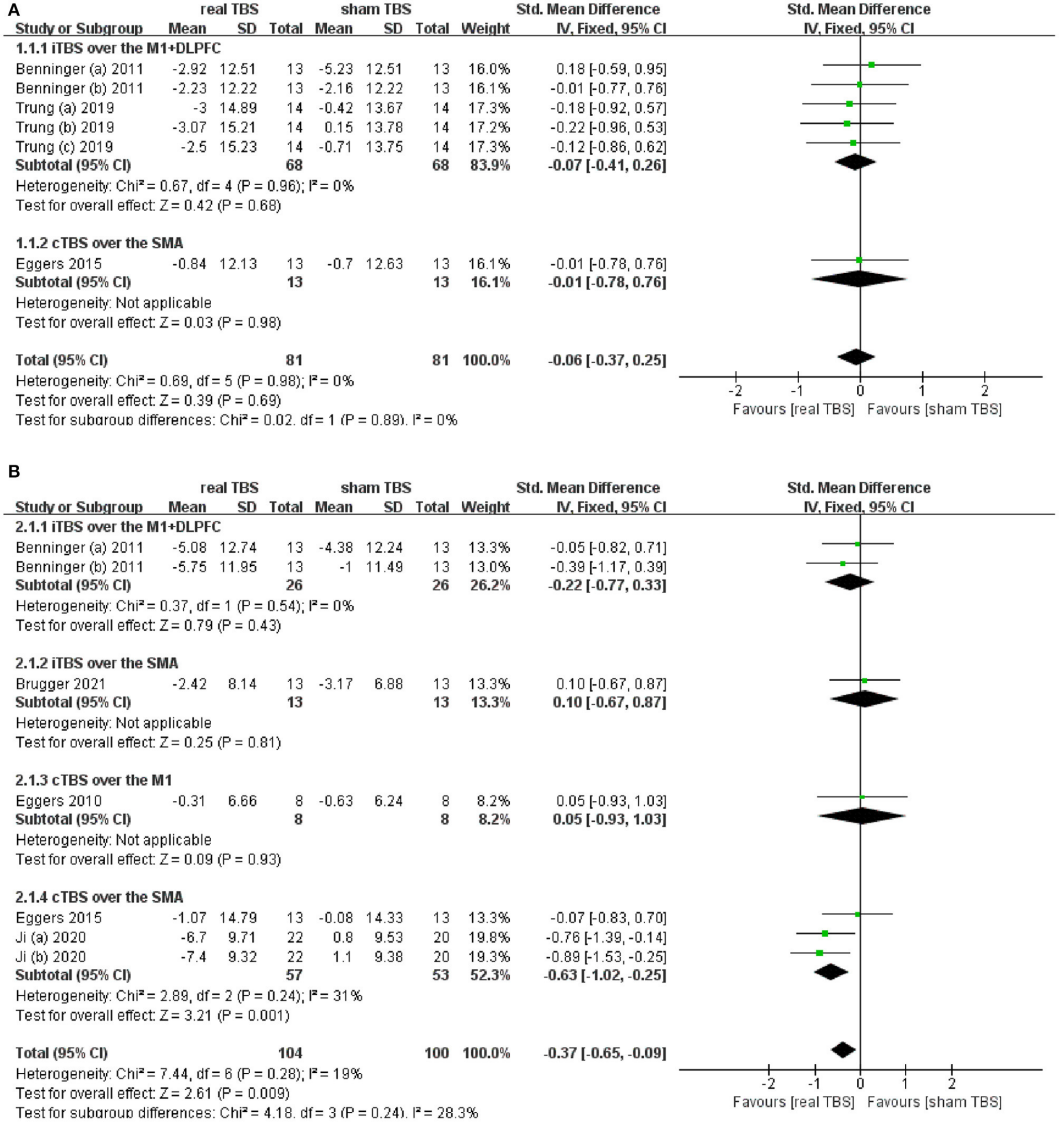
**(A)** Forest plot and metaanalysis of UPDRS-III score between real TBS and sham TBS in the “on” medicine state: subgroup analysis based on iTBS/cTBS-brain targets. **(B)** Forest plot and metaanalysis of UPDRS-III score between real TBS and sham TBS in the “off” medicine state: subgroup analysis based on iTBS/cTBS-brain targets.

Six included studies provided the date of Unified Parkinson's Disease Rating Scale part III (UPDRS-III) score in the “on” and/or “off” medicine status (23, 24, 28, 30, 32, 34). The results showed that there was an insignificant difference in UPDRS-III score between the real TBS and the sham TBS in the “on” medicine state (SMD = −0.06; 95% CI, −0.37 to 0.25; *P* = 0.69; *I*^2^ = 0%. Figure 2A). Subgroup analysis based on different types of TBS (iTBS/cTBS) over the related brain targets (iTBS/cTBS- brain targets) showed insignificant differences among groups [iTBS- M1+DLPFC, SMD = −0.07; 95% CI, −0.41 to 0.26; *p* = 0.68, vs. cTBS-supplementary motor area (SMA), SMD = −0.01; 95% CI, −0.78 to 076; *p* = 0.98, Figure 2A]. Contrarily, there was a significant difference of UPDRS-III score between real TBS and sham TBS in the “off” medicine condition (SMD = −0.37; 95% CI, −0.65 to −0.09; *p* < 0.01; *I*^2^ = 19%, Figure 2B). Further subgroup analyses based on iTBS/cTBS-brain targets showed that merely cTBS-SMA brought significant improvement of UPDRS-III score (SMD = −0.63; 95% CI, −1.02 to −0.25; *p* < 0.01).

### Effects of TBS on Upper Limb Movement

**Figure 3:**
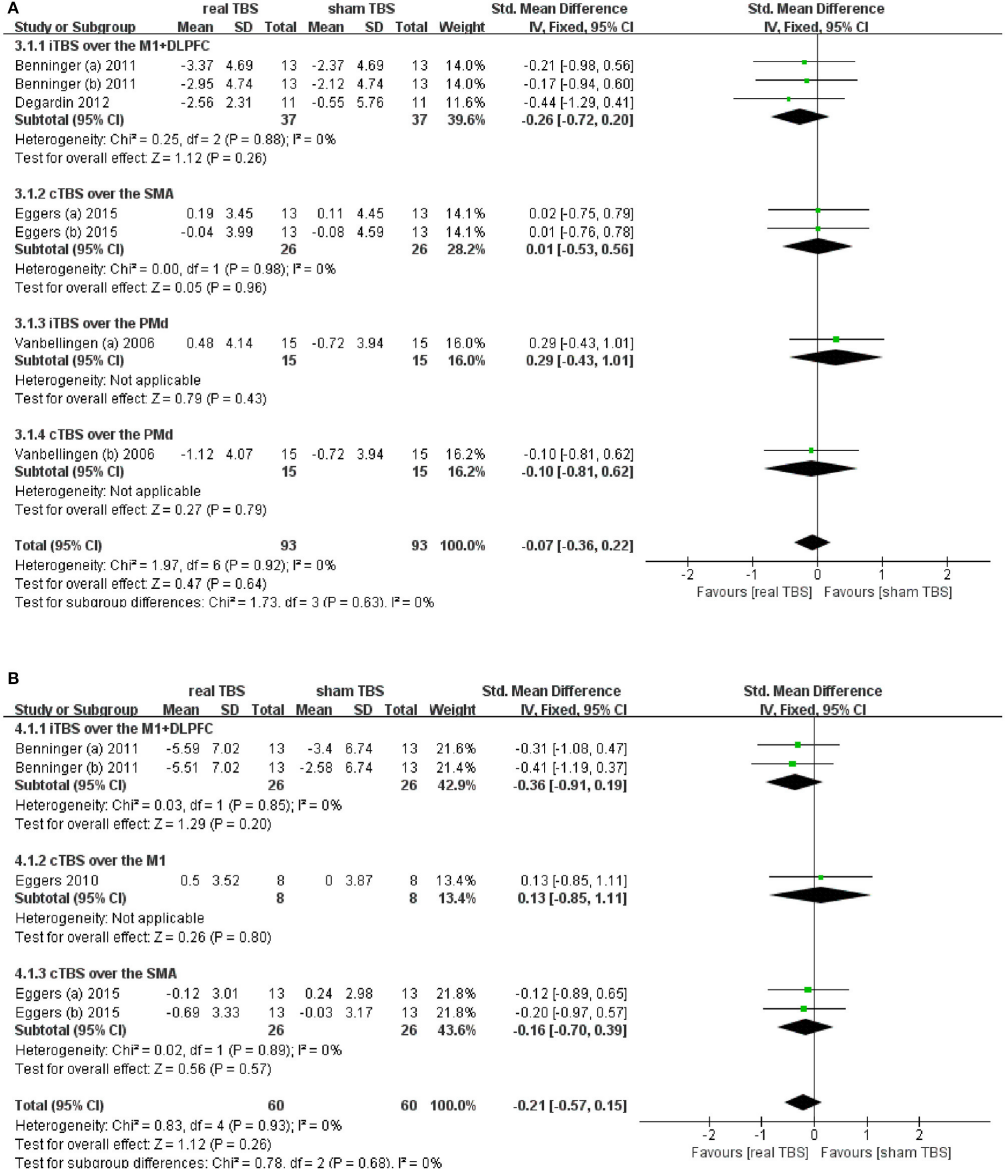
**(A)** Forest plot and metaanalysis of upper limb movement between real TBS and sham TBS in the “on” medicine state: subgroup analysis based on iTBS/cTBS-brain targets. **(B)** Forest plot and metaanalysis of upper limb movement between real TBS and sham TBS in the “off” medicine state: subgroup analysis based on iTBS/cTBS-brain targets.

Five studies reported changes in symptom scale score concerning upper limb motor dysfunction in “on” and/or “off” medicine status (23–25, 28–30). UPDRS-III bradykinesia of sequential hand and arm movement time items; UPDRS-III finger tapping, hand movement, and arm rigidity items; and Purdue Pegboard test (PPT) were involved totally. Results showed that there was an insignificant difference in the upper limb motor disorder scores between real TBS and sham TBS in the “on” medicine status (SMD = −0.07; 95% CI, −0.36 to 0.22; *p* = 0.64; *I*^2^ = 0%, Figure 3A). Subgroup analysis based on iTBS/cTBS-brain targets showed insignificant differences among groups [iTBS-M1+DLPFC, SMD = −0.26; 95% CI, −0.72 to 0.20; *P* = 0.26, vs. cTBS-SMA, SMD = 0.01; 95% CI, −0.53 to 0.56, *P* = 0.96, vs. iTBS- dorsal premotor cortex (PMd), SMD = 0.29; 95% CI, −0.43 to 1.01, *p* = 0.43, vs. cTBS-PMd, SMD = −0.10; 95% CI, −0.81 to 0.62; *P* = 0.79. Figure 3A]. Similarly, there was no significant difference in the “off” medicine status (SMD = −0.21; 95% CI, −0.57 to 0.15; *P* = 0.26; *I*^2^ = 0%, Figure 3B). Subgroup analysis based on iTBS/cTBS-brain targets shown insignificant differences among groups (iTBS-M1+DLPFC, SMD = −0.36; 95% CI, −0.91 to 0.19; *P* = 0.20, vs. cTBS-M1, SMD = 0.13; 95% CI, −0.85 to 1.11; *P* = 0.80, vs. cTBS- SMA, SMD = −0.16; 95% CI, −0.70 to 0.39; *P* = 0.57. Figure 3B).

### Effects of TBS on Gait Disorders

**Figure 4:**
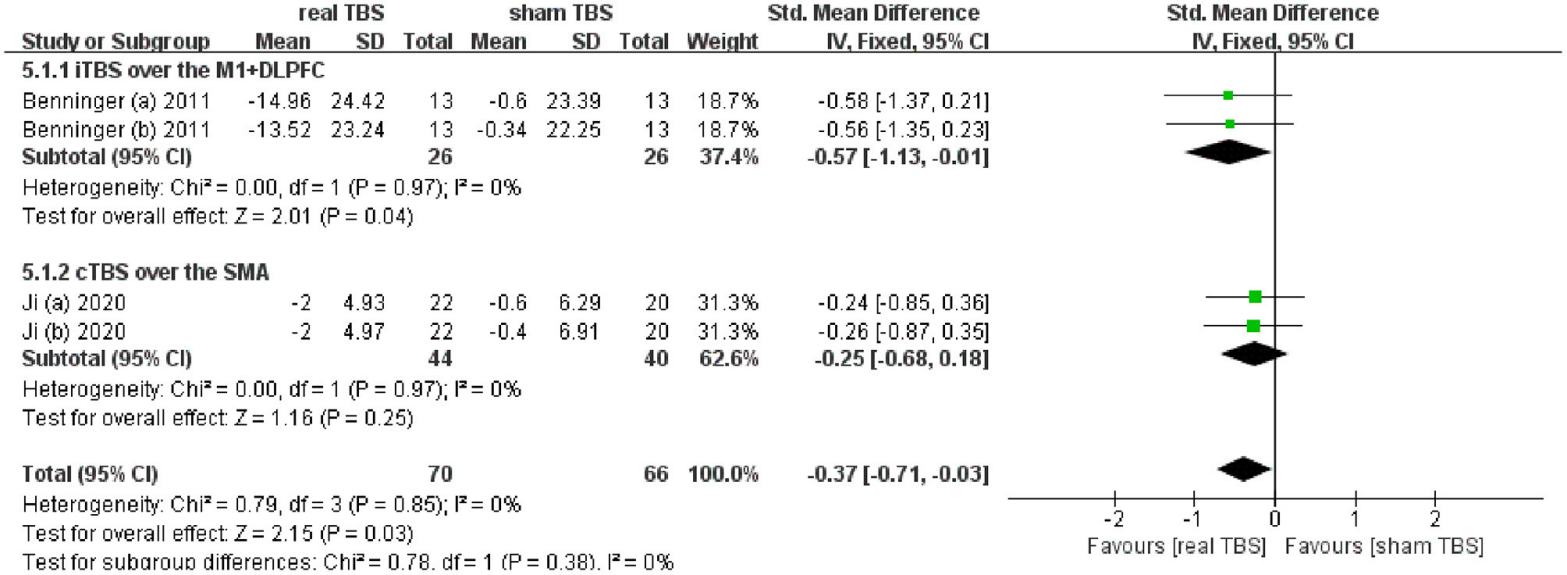
Forest plot and metaanalysis of gait disorder between real TBS and sham TBS in the “off” medicine state: subgroup analysis based on iTBS/cTBS-brain targets.

Two included studies reported the data of assessing the therapeutic effect on slowing of gait (including the time to walk 10 meters and 20 meters) (24, 32). The meta-analysis shown a significant difference of gait disorder in real TBS relative to sham TBS in the “off” medicine status (SMD = −0.37; 95% CI, −0.71 to −0.03; *P* = 0.03; *I*^2^ = 0%, Figure 4). Subgroup analysis based on iTBS/cTBS-brain targets suggested that there was significant difference between groups (iTBS-M1+DLPFC, SMD = −0.57; 95% CI, −1.13 to −0.01; *P* = 0.04, vs. cTBS-SMA, SMD = −0.25; 95% CI, −0.68 to 0.18; *p* = 0.25, Figure 4).

### Effects of TBS on Depression

**Figure 5:**
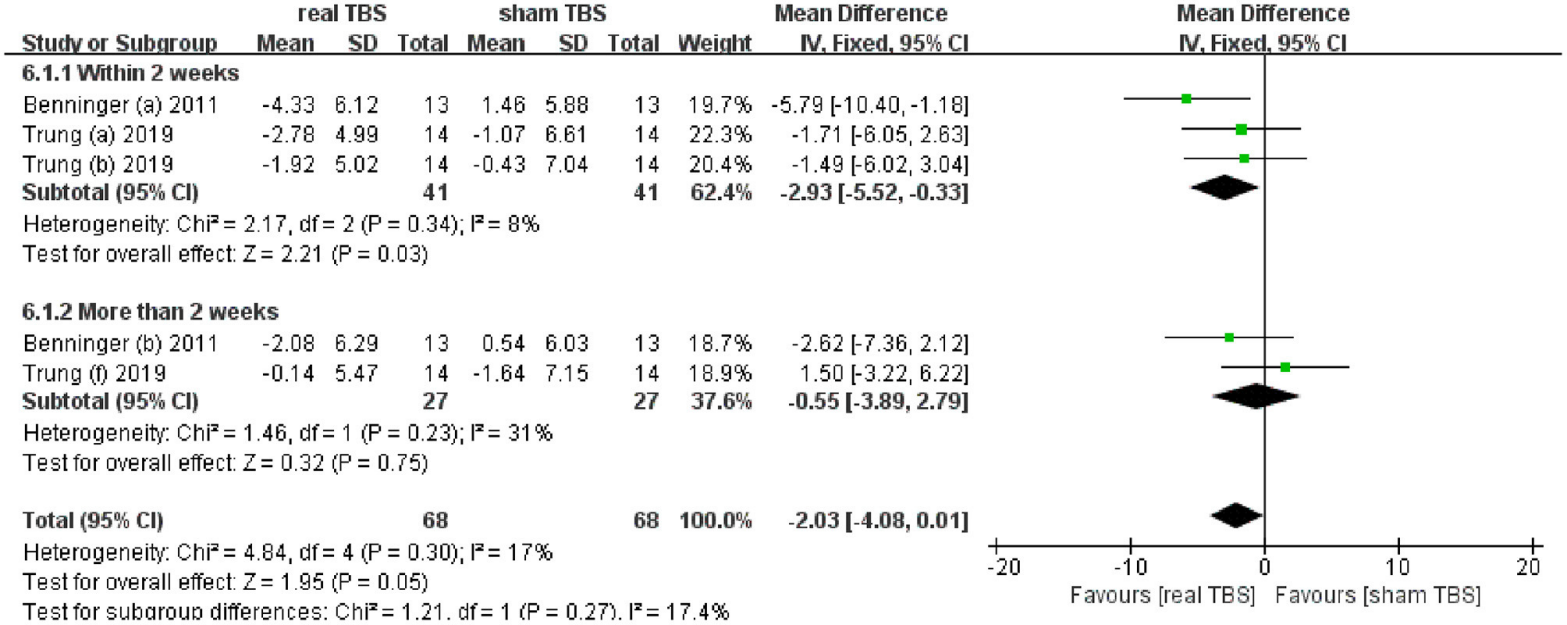
Forest plot and metaanalysis of depression between real TBS and sham TBS: subgroup analysis based on different follow-up times.

Two included studies reported the date of antidepressant effect by assessing beck depression inventory (BDI) scores (24, 30). The metaanalysis showed no significant difference in BDI scores for real TBS relative to sham TBS (MD = −2.03; 95% CI, −4.08 to 0.01; *p* = 0.05; *I*^2^ = 17%, Figure 5). Subgroup analysis based on different follow-up time showed that there was a significant difference between groups (within 2 weeks, MD = −2.93; 95% CI, −5.52 to −0.33; *p* = 0.03, vs. more than 2 weeks, MD = −0.55; 95% CI, −3.89 to 2.79; *p* = 0.75, Figure 5).

### Effects of TBS on Cognitive Impairment and Dementia

Four included studies explored the therapeutic effect of TBS on cognitive dysfunction in patients with PD by evaluating the scores of cognitive domain scales (30, 31, 33, 35), and the results among these studies were inconsistent. We failed to synthesize data of cognitive disorders because we can only get details of the data (mean ± SD/SE) from one of the mentioned articles.

### Safety/Adverse Events

Of the 14 studies included in this review, three studies (26, 30, 34) did not mention whether there were adverse events. Eleven studies (22–25, 27–29, 31–33, 35) reported that there were no serious adverse events, and one of the studies (33) reported uncomfortable sensation over local and adjacent areas of stimulation site during iTBS application, which was resolved by reducing the stimulation intensity (3–5%).

## Discussion

To the best of our knowledge, this is the first metaanalysis to evaluate the therapeutic effect of TBS, patterned rTMS, on motor and nonmotor symptoms in patients with PD. This study provides evidence to demonstrate that iTBS-M1+DLPFC or cTBS-SMA did not significantly decrease the UPDRS-III score in the “on” medicine state, while, cTBS-SMA, not iTBS-M1+DLPFC, iTBS-SMA, and cTBS-M1, could significantly improve the UPDRS-III score of these patients in the “off” medicine state. TBS had insignificant efficacy for upper limb movement disorder both in the “on” medicine state (iTBS-M1+DLPFC, cTBS-SMA, cTBS-PMd, and iTBS-PMd), and in the “off” medicine state (iTBS-M1+DLPFC, cTBS-M1, and cTBS-SMA). ITBS-M1+DLPFC, not cTBS-SMA, significantly improved slowing of gait in PD patients in the “off” medicine status. Additionally, iTBS -M1+DLPFC had a short-term (within 2 weeks) significant antidepressant effect on patients with PD.

The UPDRS-III is a reliable and valid scale for the assessment of motor performance for patients with PD, which also correlates with disease severity and quality of daily life (52). Our results demonstrated that TBS did not significantly improve the UPDRS-III score in the “on” medicine state. While there was a significant therapeutic effect in the withdrawal medicine state. Considering that different types of TBS (iTBS/cTBS) over the related brain targets may produce a significant difference of therapeutic effect, we performed subgroup analysis based on iTBS/cTBS-brain targets in both the “on” and “off” medicine status, which found that iTBS- M1+DLPFC or cTBS-SMA did not significantly improve UPDRS-III score in the “on” medicine state. It is unexpected that facilitatory iTBS over the M1 did not improve total motor performance (17). **Due to the limitation of the sample size in our study, the therapeutic effect of iTBS on the overall motor disorder in PD patients may be underestimated (type II error)**. Conversely, cTBS-SMA significantly improved the UPDRS-III score in the “off” medicine state. For two included studies, Ji et al. gave cTBS-SMA for consecutive 14 days, a total of 42 sessions, and the results showed that cTBS over the SMA had a significant improvement of UPDRS-III score with follow-up for 2 weeks (32), **where change of UPDRS-III score reached a level that reflects a significant clinical improvement** **(****53**, **54****)**, while in Eggers et al. work, single-session iTBS over the SMA did not significantly improve UPDRS-III score (28). Insufficient stimulation sessions may be an important reason. In our study, cTBS over the SMA improved the UPDRS-III score, which is consistent with previous studies that the improvement of motor symptoms was related to inhibiting the excitability of SMA (55–57). In terms of that cTBS-SMA significantly improved UPDRS-III in “off,” not in the “on,” medicine status, a possible mechanism is that dopamine has been suggested to have negative effects on the plasticity of the motor cortex in patients with PD (58). Most of the included studies in this meta-analysis defined “off” medicine state as anti-Parkinsonism medicine withdrawal status for at least 12 h, without considering the type of drug. It is necessary to select the scientific withdrawal time based on the different half-life of drugs and consider the equivalent dose of levodopa for confirming the difference of iTBS/cTBS over the corresponding brain targets in the “on” and “off” medicine state.

Upper limb motor dysfunction in patients with PD included a decrease concerning the speed and amplitude of movement, reaching and grasping deficits, and reduction of hand dexterity. Despite various upper limb dysfunctions that occur in PD, few studies reported treatment interventions for enhancing upper limb function (59). Our metaanalysis showed that TBS did not significantly improve the upper limb motor scores both in the “on” and “off” medicine state. Further analysis based on iTBS/cTBS-brain targets also found an insignificant difference among subgroups. PPT was chosen to explore upper limb movement and hand flexibility in two studies (23, 28), which has been demonstrated to correlate with disease severity, hand dexterity, and limitation of activity in patients with PD (60). Other studies selected the subitems of UPDRS-III concerning upper limb motor function (24, 25, 29). It is worth noting that Eggers et al. performed single-session cTBS over the SMA, and there was a significant difference in UPDRS-III score in the “off” medicine state (post-cTBS vs. pre-cTBS, *p* = 0.024), while did not have any significant effect in the “on” medicine status (28). As mentioned earlier, the possible mechanism may be the negative effect of dopaminergic drugs on the plasticity of the motor cortex. It is necessary to explore the efficacy of TBS on upper limb motor disorder in patients with PD by optimizing brain target and giving cumulative multiple-session TBS in the future.

For patients with PD, gait disorders and recurrent falls are common and cause disability in an advanced stage. Previous metaanalyses concluded modest efficacy of HF rTMS on motor performance in PD (61, 62). Controlled rTMS studies demonstrated positive effects on gait (63–65), suggesting more powerful stimulation protocols, such as TBS, could enhance efficacy (66, 67). Our results showed that TBS significantly improved the slowing of gait in PD in the “off” medicine state. Subgroup analysis based on iTBS/cTBS-brain targets found that iTBS-M1+DLPFC, not cTBS- SMA, had a significant therapeutic effect. Our results merely come from two studies (24, 32), and there was the limitation of a small sample size. In this metaanalysis, we failed to make a synthesis analysis for the date of freezing of gait (FOG). For PD patients, FOG is a refractory motor dysfunction resulting in an increased risk of falls. Two studies explored the efficacy of TBS on FOG by the gait parameter analysis (34, 68). The first study found that single-session iTBS- left premotor cortex did not improve FOG under normal medicine. The second study suggested that iTBS- SMA overall brought relative deterioration of gait, mainly in the time domain. The therapeutic effect of TBS on gait disorder in patients with PD needs to be further explored, and combined symptom scale of gaits and the gait parameter analysis are expected to be more effective for assessing improvement of gaits.

Depression is common in patients with PD, and it could even appear before the onset of motor symptoms. Previous studies regarding traditional rTMS found that HF rTMS over the DLPFC significantly improved PD depression, and the related guidelines also give recommendations for rTMS to intervene depression of patients with PD (13, 15–17). Our work found that iTBS-M1+DLPFC did not significantly improve the BDI score. However, subgroup analysis based on follow-up time found that it could bring short-term (within 2 weeks) therapeutic effect. For included two studies, Benninger et al. gave eight sessions of iTBS (within 2 weeks) over the M1+DLPFC (24), and Trung et al. performed six sessions of iTBS (within 1 week) over the left DLPFC (30). Whether more sessions and further optimized intervention targets can bring longer antidepressant effects requires further research.

Cognitive impairment and dementia are frequent in patients with PD (69). A growing number of researches support the view that cognitive decline in PD is mediated by degeneration and dysfunction of neural networks (70). Recent work assessing the efficacy of TBS in PD with mild cognitive impairment (PD-MCI) has shown mixed results. Hill et al. demonstrated that single-session iTBS-left DLPFC did not significantly improve working memory and executive function (31). Studies from Trung et al. and Lang et al. found that iTBS-left DLPFC had an insignificant effect on cognitive domain *z*-scores across time when comparing real with sham stimulation and correcting for multiple comparisons across cognitive domains (both received a total of six sessions iTBS within a week). However, the real iTBS group demonstrated a trend in the improvement of cognitive domain scores with 1-month follow-up compared with sham iTBS (30, 33). He and colleagues suggested that iTBS-left DLPFC for 10 consecutive weekdays brought significant improvement of repeatable battery for the assessment of neuropsychological status (RBANS) and Montreal cognitive assessment (MoCA) scores with a 3-month follow-up (*p* < 0.001 for both) (35). Considering that multiple-session iTBS over left DLPFC had a positive impact on cognitive scores, future research is promising.

It is worth mentioning that Bentley et al. explored the neurophysiology changes of DLPFC after iTBS on different deep brain stimulation (DBS) targets, subthalamic nucleus (STN), or globus pallidus interna (GPi) in seven patients with PD and found that GPi stimulation results in significantly greater theta power vs. STN stimulation (71). It is the first study that suggested TBS can be safely transmitted to human subcortical by DBS. A recent RCT demonstrated that TBS on DBS HF (200 Hz) and low-frequency (50 Hz) TBS with adapted stimulation amplitude were effective in the reduction of PD motor symptoms (akinesia, tremor, and rigidity) in 17 patients with PD (72), and had no serious adverse events.

### Limitations and Future Directions

Our study has several limitations. Firstly, the total number of included participants was small, as mentioned above; hence interpreting results should be cautiously done. Secondly, interpretation of changes for behavioral assessment should be associated with reaching a level that reflects a significant clinical improvement. Third, several uncontrolled variables, such as disease stage, side of onset, age, and sex, exist that could confound the results and must be acknowledged. Lastly, we did not definite the optimal iTBS/cTBS-brain targets and parameters of TBS that could bring significant therapeutic effect due to the limitation of the data in these included studies. A further study combining TBS with different neuroimaging techniques may better discover the potential pathophysiological mechanisms of clinical benefit and optimize TBS treatment protocols. Compared with the figure of eight coils mainly used in our included studies, the double-cone coil has the advantage of a stronger magnetic field with higher penetration depth, which is worthy of further study. Additionally, future research should try to establish a more precise relationship between the TBS effect and PD patients' clinical and demographic characteristics, such as anti-Parkinsonism medicine regimen, stage of disease, side of onset, symptom subtype (e.g., specific cognitive domain impairment), age, and gender, for finding the optimal stimulation protocols for individualized TBS treatment. Lastly, multicenter, large sample research is necessary for the future to evaluate the application prospect of TBS on invasive brain stimulation for expanding the therapeutic window and enhancing clinical benefits in PD.

## Conclusion

Our study demonstrated that cTBS-SMA could significantly improve the UPDRS-III score for patients with PD in the “off,” not in the “on,” medicine state, whereas TBS could not bring significant benefits to upper limb movement. ITBS- M1+DLPFC could significantly improve the slowing of gait in the “off” medicine status. Additionally, iTBS- M1+DLPFC has a short-term (within 2 weeks) therapeutic effect on PD depression. Since the limitations, such as small sample size and heterogeneity of assessment scale among included studies, further researches of a large sample, comprehensive evaluation, and multi-center excellent-designed RCTs are needed to confirm our research conclusions.

## Data Availability Statement

The original contributions presented in the study are included in the article/supplementary material, further inquiries can be directed to the corresponding author.

## Author Contributions

SZ developed the review concept. BC and TZ performed the data collection, analysis, and interpretation under the supervision of SZ and WZ. LS, YS, and WX contributed reagents, materials, and analysis. BC wrote the manuscript. All authors approved the final version of the manuscript for submission.

## Funding

This work was supported by the Science Foundation of the Affiliated Hospital of North Sichuan Medical College (Grants No. 2019ZD014), the Primary Health Development Research Center of Sichuan Province Program (Grants Nos. SWFZ17-Z-13; SWFZ21-Y-29), and the Initial Scientific Research Foundation of Doctors of North Sichuan Medical College (Grants No. CBY16-QD08).

## Conflict of Interest

The authors declare that the research was conducted in the absence of any commercial or financial relationships that could be construed as a potential conflict of interest.

## Publisher's Note

All claims expressed in this article are solely those of the authors and do not necessarily represent those of their affiliated organizations, or those of the publisher, the editors and the reviewers. Any product that may be evaluated in this article, or claim that may be made by its manufacturer, is not guaranteed or endorsed by the publisher.
